# Ewing-like sarcoma/undifferentiated round cell sarcoma in an infant with APC and MSH6 variation: A case report

**DOI:** 10.1097/MD.0000000000017872

**Published:** 2019-11-11

**Authors:** Jieni Xiong, Kun Zhu, Junqing Mao, Jiabin Cai, Min He, Linjie Li, Jinhu Wang, Larry Wang

**Affiliations:** a Department of Surgical Oncology; b Department of Pathology, Children's Hospital of Zhejiang University School of Medicine. Hangzhou, China; c Pathology & Laboratory Medicine, Children's Hospital Los Angeles, Los Angeles, CA.

**Keywords:** *APC*, Ewing-like sarcoma/undifferentiated round cell sarcoma, extremity, infancy, *MSH6*

## Abstract

**Rationale:**

Ewing-like sarcoma (ELS)/undifferentiated round cell sarcoma (URCS) is a rare type of soft tissue sarcomas (STS), especially in infants, with poor prognosis. It is a so-called “small round cell” sarcoma, and has many features of Ewing sarcoma, but lacks rearrangements in *EWSR1.* The diagnosis and treatment of this kind of STS remains challenging. *BCOR* genetic abnormalities have been found in some Ewing-like sarcomas.

**Patient concerns:**

This report presents an ELS case of a female infant, who was 2 months old when initially diagnosed, with the clinical stage of IIIA (G_2_T_2_N_0_M_0_). Histologic findings revealed an undifferentiated neoplasm composed of small round tumor cells with round, open chromatic nuclei, and scant cytoplasm in a sheet growth pattern. Fluorescence in situ hybridization (FISH) analysis showed absence of *EWSR1* and *ETV6* gene rearrangement. Molecular genetic testing found no established variants of clinical significance but variants of unknown significance in *APC*, *KMT2D*, and *MSH6* were detected. Immunostaining revealed that the tumor cells were positive for TLE1 and BCOR, and negative for cytokeratin (AE1/AE3), Desmin, CD45, S100, CD31, HMB45, and SATB2. INI-1 was retained.

**Diagnosis:**

Ewing-like sarcoma (ELS)/undifferentiated round cell sarcoma (URCS)

**Interventions:**

After initial diagnosis, the patient received 4 cycles of combination chemotherapy for 2 months. Radical amputation of left upper extremity was performed 3 months after diagnosis. Postoperative chemotherapy was continued for 6 cycles.

**Outcomes:**

The patient died of intracranial metastasis with hemorrhage in 13 months after initial diagnosis, 5 months after the last cycle of chemotherapy.

**Lessons:**

ELS in infancy is extremely rare and has a poorer prognosis than Ewing sarcoma or infantile fibrosarcoma. *APC* and *MSH6* variation might be related with the disease progression and predict a poorer prognosis. This rare case promotes better understanding of the disease and suggests a promising role for the combination chemotherapy regimen in treating infantile ELS. Importantly, it brings to light the possibility of intracranial metastasis, which requires proactive screening for timely detection.

## Abstract 2

Supplemental Digital Content is available in the text

## 1 Introduction

The incidence of soft tissue sarcoma (STS) in infants (age <1 year) is rare. While infantile fibrosarcoma (IFS) is the most common tumor at the extremities and rhabdomyosarcoma is the most common at all sites,^[1]^ Ewing-like sarcoma (ELS)/undifferentiated round cell sarcoma (URCS) occurs rarely during infancy or even in older children.

ELS/URCS refers to a kind of “small round cell” sarcoma, with many features of Ewing sarcoma, but lacks rearrangements in the EWSR1 gene, a salient characteristic of Ewing sarcoma.^[2,3]^ ELS/URCS is usually more aggressive than Ewing sarcoma.^[4]^ and has recently been associated with two recurrent oncogenic fusion rearrangements, BCOR-CCNB3 and CIC-DUX4 in the literature.^[4–10]^

An extremely rare case of aggressive ELS in the left forearm of a 2-month-old infant with BCL6 corepressor (BCOR) expression is presented in this report. The challenges associated with diagnosis, the factors related to poor prognosis and unique results of molecular testing are discussed in this case report.

## 2 Case presentation

The patient's parents have signed informed consent for publication of the case report and any accompanying images.

**Figure 1:**
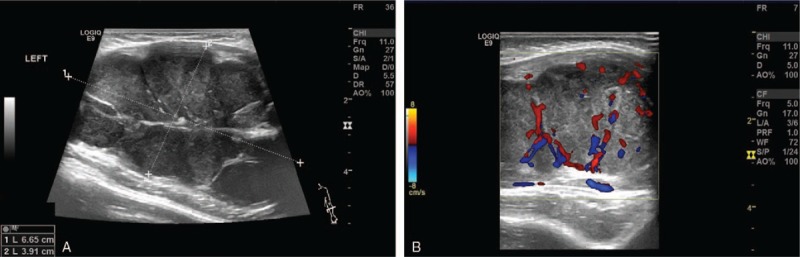
B-scan ultrasonography a 7.8 cm × 3.8 cm × 4.3 cm hypoechoic mass in the muscle layer with clear boundary and lobulated shape; Color Doppler flow imaging (CDFI) showed abundant blood flow signals in the mass.

**Figure 2:**
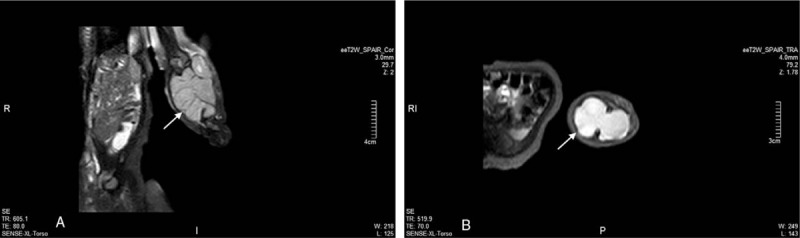
Magnetic resonance (MR) imaging revealed a solid subcutaneous neoplasm with long T1 and T2 signals in the left forearm which was partially deep between the ulnar and humerus (Arrow).

A 2-month-old infant girl was admitted in January 2018 after presenting with a rapidly growing mass in the left forearm, which her parents detected in the previous month. The infant was otherwise healthy; she had no prenatal abnormalities and displayed appropriate growth and development for her age. Members of her family did not suffer from a similar disease or malignant tumors. On physical examination, a large, firm, solid subcutaneous mass measuring 8.0 cm × 5.0 cm × 4.0 cm in size was palpable in the left forearm, with no apparent change in the skin or deficiency in limb activity. Color Doppler flow imaging (CDFI) showed a 7.8 cm × 3.8 cm × 4.3 cm hypoechoic mass in the muscle layer with lobulated shape and abundant blood flow signals (Fig. 1). Magnetic resonance (MR) imaging revealed a solid subcutaneous neoplasm in the left forearm which was partially deep between the ulnar and humerus (Fig. 2).

**Figure 3:**
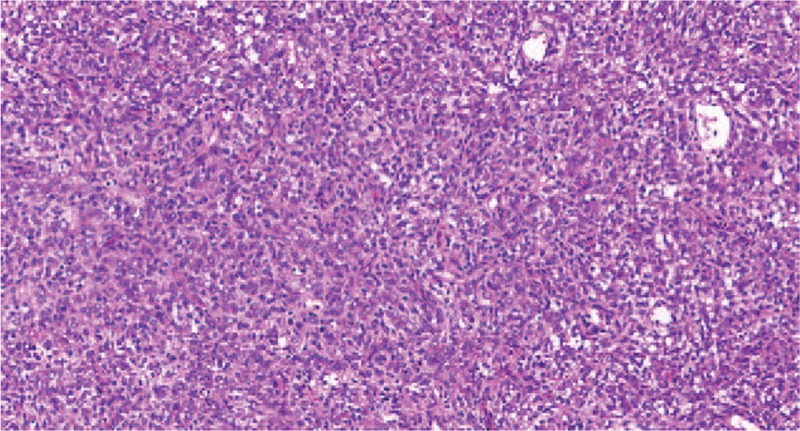
Microscopy (H&E staining, × 200) showed undifferentiated neoplasm composed of small round tumor cells with round, open chromatic nuclei, and scant cytoplasm in a sheet growth pattern. Mitosis was present (up to 3/10 HPF). Scattered apoptotic cells were present.

**Figure 4:**
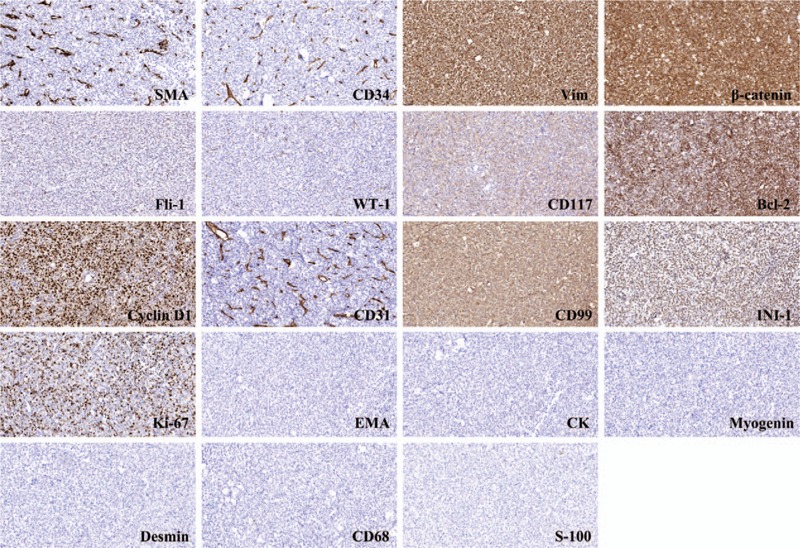
Immunohistochemistry (IHC, × 200) revealed that the tumor cells were positive for SMA, CD34, Vim, β-catenin, Fli-1, WT-1, CD117, Bcl-2, CyclinD1, CD31, CD99, INI-1, Ki-67 (80%), while negative for EMA, CK, Myogenin, Desmin, CD68, and S-100.

Due to the large size and suspected malignancy of the tumor, an open incisional biopsy was performed. The histological findings showed an undifferentiated neoplasm composed of small round tumor cells with round, open chromatic nuclei, and scant cytoplasm in a sheet growth pattern (Fig. 3). Immunohistochemistry (IHC) revealed that the tumor cells were positive for SMA, CD34, Vim, β-catenin, Fli-1, WT-1, CD117, Bcl-2, CyclinD1, CD31, CD99, INI-1 and Ki-67 (80%), and lacked EMA, CK, Myogenin, Desmin, CD68, and S-100 (Fig. 4). Fluorescence in situ hybridisation (FISH) analysis confirmed the absence of *EWSR1* and *ETV6* gene rearrangement. While molecular genetic testing (OncoKids^[11]^ Cancer Panel) showed no established variants of clinical significance, it detected variants of unknown significance detected in *APC*, *KMT2D*, and *MSH6* (Table 1). Further, immunostaining of tumor cells was positive for TLE1 and BCOR, and negative for cytokeratin (AE1/AE3), Desmin, CD45, S100, CD31, HMB45, and SATB2. INI-1 was retained (for details see supplementary material). Based on these results, a tentative diagnosis of ELS/URCS was made. Subsequent systematic evaluation (including lung CT scan, abdomen B-scan ultrasonography and radiography scan of the limbs) revealed no evidence of any other primary lesion or distant metastasis. Based on the American joint committee on cancer (AJCC) staging system for soft tissue sarcoma of the trunk and extremities (8th ed, 2017), the primary stage of the tumor was determined to be stage IIIA (G2T2N0M0).

**Table 1:**
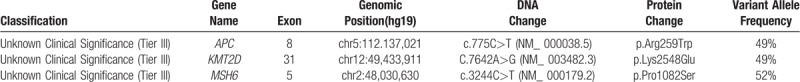
Variants of unknown significance (DNA) detected in molecular genetic testing.

**Figure 5:**
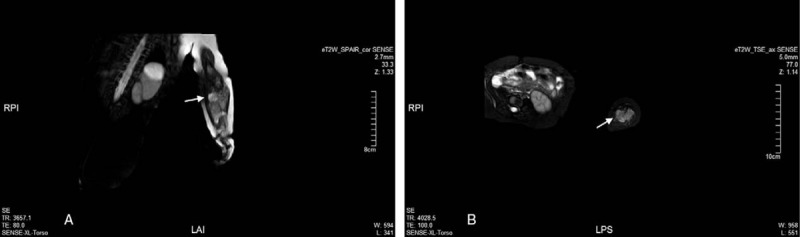
Magnetic resonance (MR) imaging revealed that there was a significant reduction in tumor size, from 7.8 cm × 3.8 cm × 4.3 cm to 3.6 cm × 2.4 cm × 2.9 cm (Arrow).

**Figure 6:**
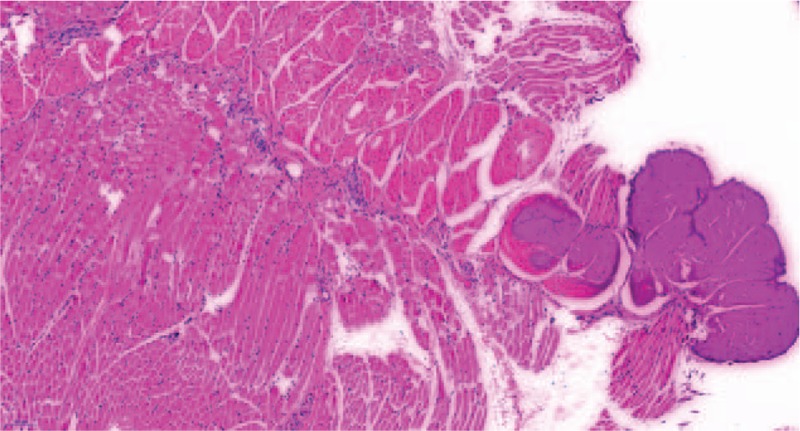
Microscopy (H&E staining, × 200) of the surgical specimen revealed a tumor residual cavity with surrounding fibrosis and hyaline degeneration. The resection margin was tumor free.

Chemotherapy was commenced within 2 weeks of the biopsy according to the NCCN guidelines for extremity/superficial trunk, head/neck (Version 1.2018). Regimen of VDC (vindesine, pirarubicin substitute for doxorubicin and cyclophosphamide) and IE (ifosfamide and etoposide) were given alternatingly at an interval of 3 weeks (Table 2) resulting in a significant reduction in tumor size (from 7.8 cm × 3.8 cm × 4.3 cm to 3.6 cm × 2.4 cm × 2.9 cm, Fig. 5), after 4 cycles of chemotherapy. A radical amputation of the left upper extremity was performed. While a tumor residual cavity with surrounding fibrosis and hyaline degeneration was observed microscopically, no residual tumor tissue was identified, and the resection margin was tumor free (Fig. 6).

**Table 2:**
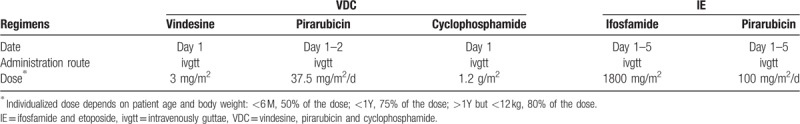
Chemotherapy regimen (VDC alternating with IE) for patient of Ewing-like sarcoma according to NCCN Guidelines.

**Figure 7:**
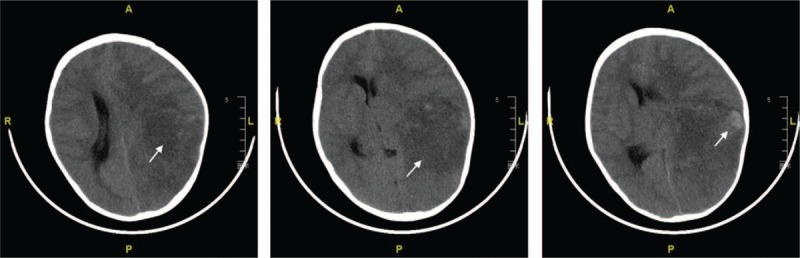
Cranial plain CT scan at the local hospital displayed a large low-density lesion with no clear boundaries in the left frontal, temporal and parietal, part of which was high density (Arrow). The left ventricle was pressed, and the midline shifted to the right.

Chemotherapy was reinitiated 1 week after surgery. After 6 cycles of postoperative chemotherapy, monthly follow-ups with the patient were conducted in the outpatient department. Unfortunately, the patient succumbed unexpectedly five months after the treatment at a local hospital because of intracranial metastases with hemorrhage (Fig. 7). The total clinical course lasted approximately 13 months, indicating that the tumor was extremely aggressive.

## 3 Discussion

Soft tissue sarcomas (STS) occurring in infants (age <1 year) represent a rare entity. Infantile fibrosarcomas (IFS) represent approximately 5% to 10% of all sarcomas in infants, and have relatively good prognosis, the 5-year overall survival (OS) rate being 89%.^[12]^ IFS frequently involve the soft tissue of the trunk and distal extremities. The clinical course is characterized by a rapidly growing large soft tissue mass that rarely metastasizes. Histologically, it demonstrates a widely variable morphology and is characterized by t(12;15) (p13;q25) translocation with *ETV6-NTRK3* gene fusion.^[13]^ In this case, based on the age of the patient and the manifestation of the tumor, the initial diagnosis was that of infantile fibrosarcoma. However, the biopsy pathology showed no evidence of IFS, neither morphologically, nor through genetic testing. On the contrary, the pathological findings revealed a rare kind of soft tissue sarcoma, an ELS/URCS. This tumor was similar to Ewing sarcoma in morphology but different in protein expression as determined by immunohistochemistry and had a poorer prognosis than IFS.^[1]^

Molecular genetic testing using OncoKids Cancer Pane revealed no established variants of clinical significance, but some variants of unknown significance including *APC*, *KMT2D*, and *MSH6* were found. Variants of unknown significance were detected in *APC* (p.Arg259Trp), *KMT2D* (p.Lys2548GIu), and *MSH6* (p.Pro1082Ser). These variants are missense alterations whose impact on protein function is uncertain. Immunostaining showed TLE1 and BCOR positive tumor cells. BCOR overexpression is a highly sensitive marker in round cell sarcomas with *BCOR* genetic abnormalities,^[5]^ which led to the final diagnosis of ELS/URCS.

ELS/URCS is a heterogenous group of tumors composed of tumor cells with monomorphic round nuclei and scant cytoplasm. It usually lacks a known recurrent genetic abnormality and remains unclassified by currently established tumor entities.^[2]^ Recently, 2 recurrent oncogenic fusion rearrangements, *BCOR-CCNB3* and *CIC-DUX4* have been described in the literature,^[4–10]^ but reports on infantile ELS/URCS are scarce. But literature about infantile ELS/URCS was rare. According to one report, recurrent *BCOR* exon 16 internal tandem duplication (ITD) and *YWHAE-NUTM2B* fusions occur in half of infantile soft tissue URCS.^[14]^ The clinicopathologic features of 22 cases are presented in Table 3, and *BCOR*-ITD was detected in 27% (6/22) of those cases. In our case, molecular testing was not designed to detect BCOR alterations, but BCOR expression was detected through immunostaining, and it is possible that this ELS case may have *BCOR*-rearrangement.

**Table 3:**
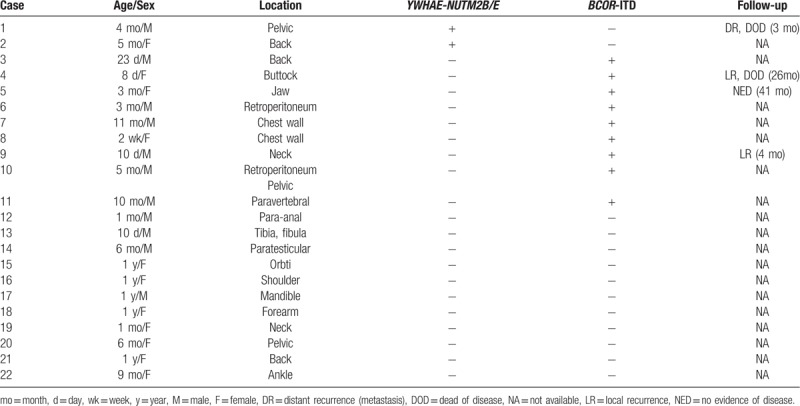
The clinicopathologic features and genetic abnormalities of infantile undifferentiated round cell sarcomas^[14]^.

Currently, there is no unified protocol for ELS. Surgery is a critical component in the treatment of all pediatric soft tissue sarcoma patients. The role for chemotherapy in the treatment of children who have soft tissue sarcoma remains unclear.^[15]^ Additionally, although radiotherapy (pre- and postoperative) is recommended for soft tissue sarcomas in adults, little is known about its benefits in infants.^[16]^ In this case, radiotherapy was not adopted and the chemotherapy regimen (VDC alternating with IE) had proved effective. Significant reduction of the primary lesion volume and a tumor residual cavity with fibrous hyperplasia and hyaline degeneration in postoperative pathology after 4 cycles of chemotherapy were observed.

Lung is a preferred metastatic organ of undifferentiated round cell sarcoma, while intracranial metastasis is rare. It is unknown if our patient had an intracranial metastasis at presentation. Despite the significant effect of neoadjuvant chemotherapy on the primary lesion, the patient died of intracranial metastasis within 13 months of diagnosis. In hindsight, the intracranial metastasis was extremely aggressive and its detection required better surveillance.

Although Ewing-like sarcomas are more aggressive than Ewing sarcoma,^[4]^ patients display longer survival with *BCOR-CCNB3* Ewing-like sarcomas in the extremities.^[17]^ Although lacking established variants of clinical significance, immunostaining revealed BCOR positive tumor cells, suggesting that this could be a case of BCOR sarcoma. However, the aggressive phenotype of the tumor was inconsistent with this presumption.

Additionally, some other variants of unknown significance were detected such as *APC*, *KMT2D*, and *MSH6*. Alterations in the APC gene have been previously detected in some cases of synovial sarcoma and were thought to contribute to the accumulation of β-catenin.^[18]^ Likewise, the relationship between *MSH6* expression and metastasis, response to chemotherapy, and survival time in patients with osteosarcoma have been investigated.^[19]^ These studies indicate a potential role for *APC* and *MSH6* mutations in sarcomas, where they may correlate with poor prognosis.

In summary, Ewing-like sarcomas of infancy are quite rare and have a poorer prognosis than Ewing sarcomas or infantile fibrosarcomas. Though some genetic abnormalities such as *CIC-DUX4* and *BCOR-CCNB3* have been investigated in recent years, the genetic characteristics of Ewing-like sarcomas are still undefined. *APC* and *MSH6* variation might be related with the disease progression and its poor prognosis. Further research is urgently needed to define the characteristics and advance the treatment of this group of rare tumors.

## Author contributions

**Conceptualization:** Jieni Xiong.

**Investigation:** Kun Zhu.

**Methodology:** Kun Zhu.

**Resources:** Junqing Mao, Jiabin Cai, Min He, Linjie Li.

**Supervision:** Jinhu Wang, Larry Wang.

**Writing – original draft:** Jieni Xiong.

**Writing – review & editing:** Jinhu Wang, Larry Wang.

## Supplementary Material

Supplemental Digital Content
