# Supplementary material for: Ewing-like sarcoma/undifferentiated round cell sarcoma in an infant with APC and MSH6 variation: A case report
Source: Medicine (Baltimore). 2019 Nov 11;98(45):e17872. doi: 10.1097/MD.0000000000017872 (PMC6855612; doi:10.1097/MD.0000000000017872)
Supplement: Supplemental Digital Content [file medi-98-e17872-s001.pdf]

Supplementary material: OncoKids Cancer Panel

CLINICAL HISTORY / INDICATION FOR TEST

Undifferentiated Sarcoma, suggestive of undifferentiated round cell sarcoma of infancy; BCOR positive by immunostain

RESULTS

VARIANTS OF UNKNOWN SIGNIFICANCE (DNA)

Classification	Gene Name	Exon	Genomic Position(hg19)	DNA Change	Protein Change	Variant Allele Frequency
Unknown Clinical Significance (Tier III)	APC	8	chr5:112.137,021	c.775C>T (NM_000038.5)	p.Arg259Trp	49%
Unknown Clinical Significance (Tier III)	KMT2D	31	chr12:49,433,911	C.7642A>G (NM_003482.3)	p.Lys2548Glu	49%
Unknown Clinical Significance (Tier III)	MSH6	5	chr2:48,030,630	c.3244C>T (NM_000179.2)	p.Pro1082Ser	52%

INTERPRETATION

No established variants of clinical significance were detected (includes gene amplifications, gene fusions, substitutions, insertions, and deletions).

Of note, this test is not designed to detect BCOR internal tandem duplications and YWHAE-NUTM2B fusions, which are recurrent alterations in undifferentiated round cell sarcoma of infancy [1,2].

Variants of Unknown Significance

Variants of unknown significance were detected in APC (p.Arg259Trp), KMT2D (p.Lys2548GIu), and MSH6 (p.Pro1082Ser). These variants are missense alterations whose functional impact to the protein is uncertain. Additionally, these variants have not been reported in sarcoma, and the role of these genes in sarcoma has not been established. This test cannot definitively distinguish between somatic and germline variants. Thus, these variants may be of germline origin.

REFERENCES

1. Kao YC, Sung YS, Zhang L, Jungbluth AA, Huang SC, Argani P, Agaram NP, Zin A, Alaggio R, Antonescu CR. BCOR overexpression is a highly sensitive marker in round cell sarcomas with BCOR genetic abnormalities. The American Journal of Surgical Pathology. 2016 Dec;40(12):1670.
2. Kao YC, Sung YS(Zhang L, Huang SC, Argani P, Chung CT, Graf NS, Wright DC, Kellie SJ, Agaram NP, Ludwig K. Recurrent BCOR Internal Tandem Duplication and YWHAE-NUTM2B Fusions in Soft Tissue Undifferentiated Round Cell Sarcoma of Infancy-Overlapping Genetic Features with Clear Cell Sarcoma of Kidney. The American Journal of Surgical Pathology. 2016 Aug;40(8):1009.

GENE LIST (DNA)

Mutation hotspots in the following genes (82) were analyzed:

ABL1, ABL2, ACVR1, AKT1, ALK, ASXL1, ASXL2, BRAF, CALR, CBL, CCND3, CCR5, CDK4, CIC, CREBBP, CRLF2, CSF1R, CSF3R, CTNNB1, DAXX, DNMT3A, EGFR, EP300, ERBB2, ERBB3, ERBB4, ESR1, EZH2, FASLG, FBXW7, FGFR2, FGFR3, FLT3, GATA2, GNA11, GNAQ, H3F3A, HDAC9. HIST1H3B, HRAS, IDH1, IDH2, IL7R, JAK1, JAK2, JAK3, KDM4C, KDR, KIT, KRAS, MAP2K1, MAP2K2, MET, MPL, MSH6, MTOR, NCOR2, NOTCH1, NPM1, NRAS. NT5C2, PAX5, PDGFRA, PDGFRB, PIK3CA, PIK3R1, PPM1D, PTPN11, RAF1, RET. RHOA, SETBP1, SETD2, SH2B3, SH2D1A, SMO, STAT3, STAT5B, TERT, TPMT, USP7, ZMYM3

Full length coding regions of the following genes (44) were sequenced:

APC, ARID1A, ARID1B, ATRX, CDKN2A, CDKN2B, CEBPA, CHD7, CRLF1, DDX3X, DICER1, EBF1, EED FAS, GATA1, GATA3, GNA13, ID3, IKZF1, KDM6A, KMT2D, MYOD1, NF1, NF2, PHF6, PRPS1, PSMB5, PTCH1, PTEN RB1, RUNX1, SMARCA4, SMARCB1, SOCS2, SUFU, SUZ12, TCF3, TET2, TP53, TSC1. TSC2, WHSC1, WT1, XIA

High-level amplification events were assessed in the following genes (24):

ALK, BRAF, CCND1, CDK4, CDK6, EGFR, ERBB2, ERBB3, FGFR1, FGFR2, FGFR3, FGFR4. GLI1, GLI2 IGF1R KIT. KRAS. MDM2, MDM4, MET, MYC, MYCN, PDGFRA, PIK3CA

GENE LIST (FUSIONS)

Fusions were analyzed from the following genes (70):

ABL1, ABL2, ALK, BCL11B, BCOR, BCR, BRAF, CAMTA1, CCND1, CIC, CREBBP, CRLF2, CSF1R, ETV6, EWSR1, FGFR1, FGFR2, FGFR3, FLT3, FUS, GUS2, JAK2, KAT3A, KMT2A, KMT2B, KMT2C, KMT2D (MLL), MAMLD1, MECOM, MET, MKL1, MLLT10, MYB. MYH11, MYH9, NCOA2, NOTCH1, NOTCH2, NPM1, NRA3, NTRK1, NTRK2, NTRK3, NUP98, NUP214, NUTM1, PAX3, PAX5, PAX7, PDGFB, PDGFRA, PDGFRB, PLAG1, RAF1, RANBP17, RARA, RELA, RET, ROS1, RUNX1, SS18, SSBP2, STAT6, TAL1, TCF3, TFE3, TSLP, USP6, YAP1, ZNF384

METHODOLOGY

DNA was extracted from FFPE [C18-76-A1] using a commercially available kit (Qiagen QIAamp DNA FFPE Tissue Kit). RNA was also extracted from FFPE [C18-76-A1] using a commercially available kit (Agencourt FormaPure Kit).

DNA libraries were generated using custom-designed Ampliseq primers targeting 3,069 amplicons. RNA was reverse transcribed into DNA; opposing primers were then used to interrogate 1,421 targeted fusions and the expression of 13 genes. Amplicons were sequenced using the Ion S5 sequencing system.

Single nucleotide variants (SNVs) and small insertions and deletions (<20 bp) were detected by mapping and comparing the DNA sequences with the human reference genome. The total number of DNA reads for this sample was 12,112,407 with an average depth of 4,462 and a mean read length of 121 base pairs. Approximately, 98.1% of amplicons were sequenced to a depth of at least 250X. For the RNA sequencing of this sample, there were 2,527,386 total mapped reads, and the mean read length was 107 base pairs.

Following mapping of the read data to the human genome (reference build GRCh37/hg19), single nucleotide variants with an aifele fraction greater than 6%,

insertions and deletions with a variant allele frequency greater than 10%, high-level amplifications (> 8 copies), and relevant fusions were annotated utilizing a customized variant curation tool. Relevant fusions were also annotated utilizing a commercially available variant curation tool (Ion Reporter, version 5.2). Reported variants include variants of strong clinical significance (tier I) and variants of potential clinical significance (tier II), and variants of unknown clinical significance (tier III). Benign and likely benign variants (tier IV) are not included in the report. Sequence alterations are reported according to CAP/AMP/ASCO guidelines. Within these guidelines, tier I variants require level A evidence (an FDA-approved therapy or inclusion into professional guidelines) or level B evidence (well-powered studies with expert consensus). Tier II variants require either level C evidence (FDA-approved therapies for different tumor types, investigational therapies, or multiple small published studies with some consensus) or level D evidence (preclinical trials or a few case reports without consensus). Tier III variants demonstrate no convincing published evidence of cancer association and are not observed at a significant allele frequency in the general or specific subpopulation databases, or pan-cancer or tumor-specific variant databases. Tier IV variants are observed at significant allele frequency in the general or specific subpopulation databases and are not associated with cancer in the literature.

Li MM, Datto Mt Duncavage EJ, Kulkarni S, Lindeman Nl, Roy S, Tsimberidou AM, Vnencak-Jones CL, Wolff DJ, Younes Af Nikiforova MN. Standards and Guidelines for the Interpretation and Reporting of Sequence Variants in Cancer: A Joint Consensus Recommendation of the Association for Molecular Pathology, American Society of Clinical Oncology, and College of American Pathologists. The Journal of Molecular Diagnostics. 2017 Jan 31; 19(1): 4-23.
